# How daytime sleepiness affects well-being in patients with schizophrenia

**DOI:** 10.1192/j.eurpsy.2026.10516

**Published:** 2026-07-31

**Authors:** D. Njah, M. Lagha, M. Bouchendira, Y. Sellaouti, A. Zyed, W. Homri

**Affiliations:** Psychiatry c, Razi psychiatric Hospital, Manouba, Tunisia

## Abstract

**Introduction:**

Daytime sleepiness (DS) is a common and underestimated symptom in patients with schizophrenia (Reeve *et al.* PLOS ONE 2021;16(1)), with a prevalence of up to 32% according to literature. It can result from medication side effects, underlying sleep disorders, or psychiatric comorbidities, such as depression. DS has a negative impact on quality of life and increases symptom severity in this population (Fujii *et al.* Sleep Biol Rhythms 2025; 23 75‑84.).

**Objectives:**

Our objective was to assess the impact of DS on the quality of life (QoL) of patients diagnosed with schizophrenia and schizoaffective disorder (SAD).

**Methods:**

This was a cross-sectional study including patients with schizophrenia and SAD aged between 18 and 65, followed up at RAZI psychiatric Hospital in Tunisia. DS was assessed using the Arabic version of the Epworth Sleepiness Scale (ESS). QoL was assessed using the 12-item Short Form (SF-12) in its French version.

**Results:**

Fifty patients were included in our study. The mean age was 42 ± 12 years and 60% were male. The median ESS score was 8 [1.5; 10.2], with a maximum of 17 and a minimum of 0. The median physical health score (PCS-12) was 42.23 [31.08; 52.72], while the mean mental health score (MCS-12) was 42.91 ± 11.12. Both PCS-12 and MCS-12 scores were below the average value (50) observed in the general population. The median ESS score was 8 [1.5; 10.2]. We found that 32% of patients (n=16) did not experience daytime sleepiness, while 68% (n=34) exhibited signs of daytime sleepiness. A significant association was found between MCS-12 and ESS scores (p < 0.001), with higher levels of sleepiness associated with lower mental health scores (β = −1.648). ESS scores explained 59% of the variance in MCS-12 scores (R² = 0.596). Moreover, PCS-12 scores were also significantly associated with ESS scores (p < 0.001), with greater sleepiness linked to lower physical health scores (β = −1.771). ESS scores accounted for 61% of the variance in PCS-12 scores (R² = 0.611).

**Conclusions:**

Daytime sleepiness has a significant impact on the physical and mental health of patients with schizophrenia and SAD. Various factors may be responsible, such as medication side effects, sleep disorders and depression. It is therefore crucial to identify these underlying causes and treat them appropriately to improve patients’ quality of life and overall well-being.

**Disclosure of Interest:**

None Declared

